# Evaluation of dermatologic adverse events associated with aromatase inhibitors: insights from the FAERS database

**DOI:** 10.3389/fphar.2025.1529342

**Published:** 2025-05-14

**Authors:** Yuan-Yuan Wu, Qiong-Lian Huang, Zhan-Yang Luo, Xiao-Yun Song, You-Yang Shi, Jin-Zhou Zheng, Sheng Liu

**Affiliations:** ^1^ Institute of Chinese Traditional Surgery, Longhua Hospital, Shanghai University of Traditional Chinese Medicine, Shanghai, China; ^2^ Shanghai Pudong Hospital, Fudan University Pudong Medical Center, Shanghai, China

**Keywords:** aromatase inhibitors, dermatologic adverse events, FDA adverse event reporting system, disproportionality analysis, real-world

## Abstract

**Background:**

This study evaluates the risk of dermatologic adverse events (AEs) associated with aromatase inhibitors (AIs) through an analysis of data from the FDA Adverse Event Reporting System (FAERS).

**Methods:**

FAERS data from Q1 2004 to Q2 2024 were analyzed for dermatologic AEs related to AIs. A disproportionality analysis using reporting odds ratio (ROR) assessed AE risk, and the time to onset of these AEs was examined.

**Results:**

Out of 21,035,995 AE reports, 2,237 involved skin impairment. Sixty-one preferred terms (PTs) presented positive signals, including nail disorders, onychoclasis, and abnormal hair growth in patients on anastrozole, exemestane, or letrozole. The highest associations were with pseudo cellulitis (ROR = 57.73), anhidrosis (ROR = 48.68), and nail toxicity (ROR = 38.40). Strong associations were observed for anastrozole (ROR = 1.07, 95% confidence interval: 1.03–1.11) and exemestane (ROR = 1.1, 95% CI: 1.04–1.16), but not for letrozole. Eleven dermatologic PTs had onset times under 50 days, with the earliest at 2 days; the latest, skin ulcer, appeared at 241.5 days with exemestane.

**Conclusion:**

The findings provide substantial evidence of dermatologic AEs associated with AIs, particularly anastrozole and exemestane, emphasizing the importance of dermatologic monitoring during AI therapy and the need for further research into AI-induced dermatologic AEs.

## 1 Introduction

Aromatase, present in both gonadal and extra-gonadal tissues, catalyzes the conversion of testosterone to estrogens (Stocco, 2012; Kharb et al., 2020). Excessive estrogen levels have been implicated in various diseases, notably contributing to the high malignancy rate of breast cancer (BC) in postmenopausal women (Diamond et al., 2015; Patel, 2017; Rižner and Romano, 2023). Consequently, inhibiting estrogen synthesis or blocking estrogenic activity is a key strategy in BC treatment, underlying the development of aromatase inhibitors (AIs) (Kharb et al., 2020). AIs are a class of drugs that target the aromatase enzyme, which is responsible for converting androgens to estrogens. AIs have evolved through three generations, each improving upon the limitations of the previous. First-generation AIs, such as aminoglutethimide, were introduced in the 1970s but lacked selectivity and specificity (Buzdar et al., 2001). Second-generation AIs, like fadrozole and formestane, offered more selectivity but still had limitations in targeting aromatase efficiently (Dellapasqua and Colleoni, 2010). The third-generation AIs, including exemestane, anastrozole and letrozole, were developed to address these shortcomings, offering greater potency and specificity, and have shown efficacy in high-risk BC patients (Kharb et al., 2020). For instance, two clinical trials involving 5,738 patients demonstrated that AIs moderately reduced distant recurrences in premenopausal BC patients, leading to improved progression-free survival (PFS) across a broad population of BC patients (Francis et al., 2018; Robertson et al., 2021). However, adverse events (AEs) associated with AIs, such as cardiovascular events, dizziness, dyslipidemia, fatigue, headache, hot flushes, joint pain, muscle pain, nausea, osteoporosis, sweating, and vaginal dryness, pose significant clinical challenges, particularly for postmenopausal BC patients (Zhang et al., 2024), thus impacting the optimal use of AIs in BC therapy. Increasingly, attention has turned to the frequent occurrence of AIs’ adverse effects.

Among the AEs associated with AIs, dermatologic reactions are particularly prevalent. For example, erythematous patches, papules, and plaques have been reported (with a median onset of 2 months) in an estrogen- and progesterone-receptor-positive BC patient receiving anastrozole (Santoro et al., 2011). Among the AEs associated with AIs, dermatologic reactions are particularly prevalent. For example, erythematous patches, papules, and plaques have been reported in an estrogen and progesterone receptor-positive BC patient receiving anastrozole, with a median onset of 2 months (Sonke et al., 2018). In an international, multicenter, randomized, double-blind, placebo-controlled phase III trial with 720 BC patients receiving exemestane, rash was the second most common AE, with other skin-related reactions also reported (Pritchard et al., 2013). These findings underscore growing concerns regarding the dermatologic safety of AIs.

Given the confirmed association between dermatologic AEs and AIs such as anastrozole, exemestane, and letrozole, it is essential to clarify the relationship between specific AI agents and dermatologic AEs. The FDA Adverse Event Reporting System (FAERS), a global spontaneous reporting system, provides extensive real-world data to identify AE risk signals (Gu et al., 2023). In this chapter, we analyze standardized FAERS data to assess the potential risk of dermatologic toxicity linked to key AI agents, aiming to inform safer options for BC therapy.

## 2 Materials and methods

### 2.1 Data source

The FAERS, accessible at https://fis.fda.gov/extensions/FPD-QDE-FAERS/FPD-QDE-FAERS.html, is a global pharmacovigilance database that records AEs, medication errors, and product quality complaints (Yang et al., 2022). This system supports the monitoring of post-marketing drugs and therapeutic biological products, helping to identify emerging safety concerns.

**FIGURE 1:**
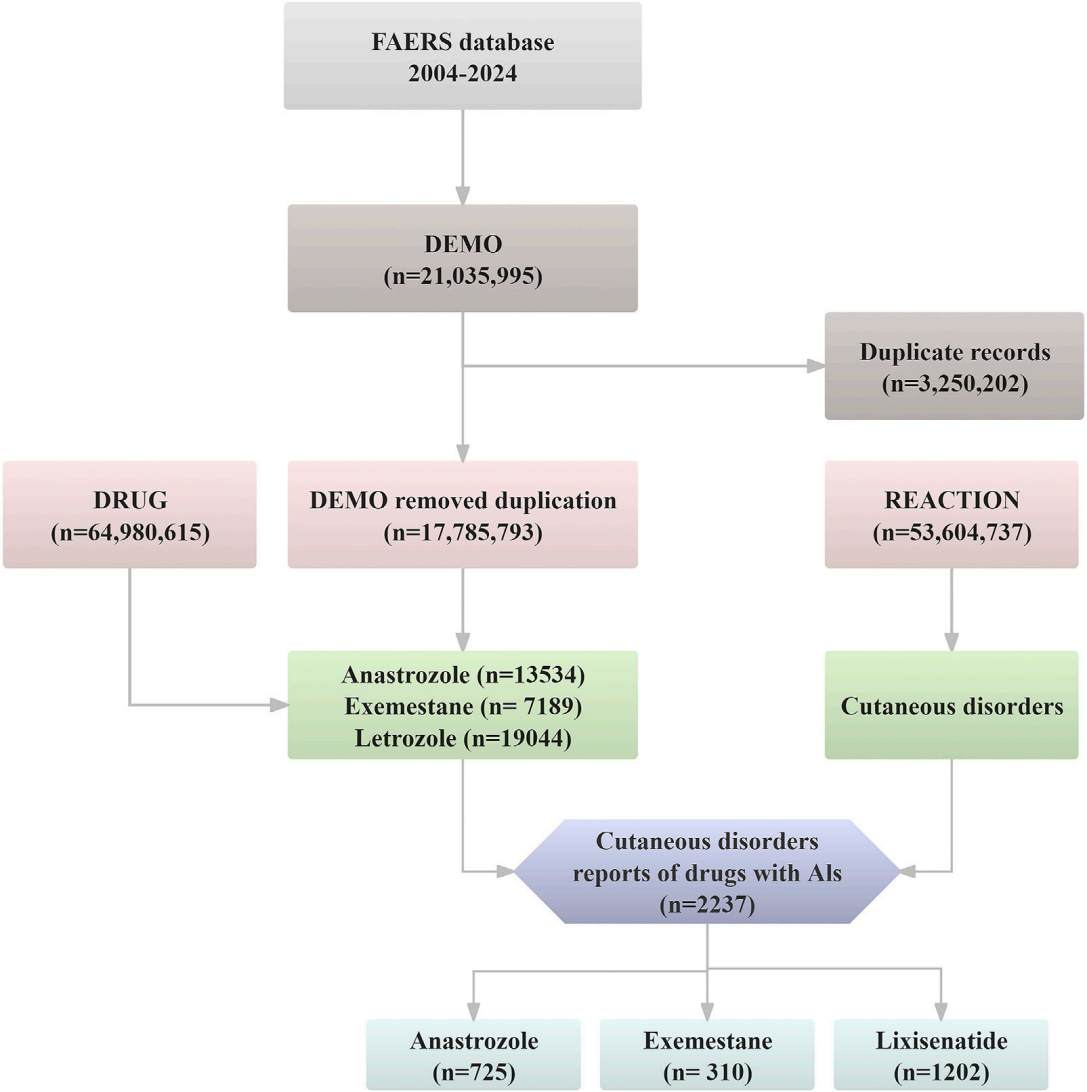
The flow diagram of confirming dermatologic AEs elicited by aromatase inhibitors from FAERS database.

The FAERS database contains 21,035,995 raw AE reports from Q1 2004 to Q2 2024. To ensure data accuracy, duplicate records were removed following the FDA-recommended deduplication process (Tregunno et al., 2014). Specifically, among records sharing the same CASEID in the DEMO table, only the report with the most recent FDA_DT was retained. If both CASEID and FDA_DT were identical, the entry with the highest PRIMARYID was preserved. After deduplication, 2,237 dermatologic AE cases related to AI agents. Further analysis focused on anastrozole (*n* = 725), exemestane (*n* = 310), and letrozole (*n* = 1,202). Figure 1 illustrates the deduplication process.

### 2.2 Data extraction

AE cases linked to three AI agents (anastrozole, exemestane, and letrozole) were identified in the FAERS DRUG file using both generic and FDA-approved brand names. Dermatologic AEs were categorized under the system organ class (SOC) code 10040785 to assess dermatologic toxicities. Additionally, the time to onset (TTO) of dermatologic AEs for each AI was calculated as the interval from treatment initiation to AE occurrence. Cases with an onset time greater than zero days were included in the analysis (Ando et al., 2019). Reports with erroneous dates (e.g., administration date after the event date) or missing dates were excluded from the dataset.

### 2.3 Signal analysis

Disproportionality analysis, also known as case/non-case analysis, is widely used in pharmacovigilance for detecting drug-adverse reaction signals (Almenoff et al., 2007). This method includes several metrics for cross-validation to enhance the robustness of signal detection and minimize false positives, such as the reporting odds ratio (ROR), proportional reporting ratio (PRR), empirical Bayes geometric mean (EBGM), and Bayesian confidence propagation neural network (BCPNN); Further statistical details are provided in Supplementary Tables S1, S2. In this study, we used ROR, a commonly accepted standard in disproportionality analysis, to identify signals of dermatologic AEs associated with AIs (Evans et al., 2001). Specifically, we calculated RORs (where the lower limit of the 95% confidence interval (CI) > 1 to evaluate the association between AIs and dermatologic AEs in FAERS data. The TTO of AIs-related AEs was calculated as the interval from AI treatment initiation (as recorded in the THER file) to AE occurrence. We modeled changes in AE incidence using the Weibull distribution (Sauzet et al., 2013).

Additionally, we performed a multivariate logistic regression model considering hospitalization, age and body weight as potential factors influencing dermatologic AEs. Incomplete reports were excluded, and categorical variables were further analyzed to identify potential risk factors.

Two authors independently conducted all data analyses, with data extraction performed using SQLiteStudio (*version 3.3.3*) and statistical analyses conducted in IBM^®^ SPSS^®^ Statistics (*version 27.0*) and R software (*version 4.3*).

## 3 Results

### 3.1 Descriptive analysis

We systematically screened and analyzed the relationship between dermatologic AEs and aromatase inhibitor (AI) agents. Reports of dermatologic AEs associated with anastrozole (*n* = 725), exemestane (*n* = 310), and letrozole (*n* = 1,202) were identified according to their respective market approval dates. Table 1 summarizes the clinical characteristics of dermatologic AEs for each AI. Notably, dermatologic AEs were significantly more common in female patients (*n* = 2,164, 96.74%) than in male patients (*n* = 17, 0.76%), with 56 cases (2.50%) of unspecified gender, likely reflecting the use of AIs primarily in postmenopausal women with BC (Francis et al., 2018). Over half of the patients had a body weight below 80 kg (*n* = 120, 50.07%), with a median weight of 68 kg. The majority of patients were over 65 years of age (*n* = 759, 33.93%), and only 1.43% were under 18 years. The median age across the cohort was 66 years, consistent across anastrozole, exemestane, and letrozole. Most AE reports were submitted by physicians (*n* = 981, 43.85%), followed by consumers (*n* = 511, 22.84%). The United States represented approximately one-third of the reports (*n* = 585, 26.15%). Among serious outcomes, important medical events were most common (*n* = 1,102, 49.26%), followed by hospitalization (*n* = 504, 22.53%).

**TABLE 1:** Characteristics of reports involved dermatologic AEs of AIs from the FAERS database. (from Q1 2004 to Q1 2024).

Characteristics	All (*n* = 2,237)	Anastrozole (*n* = 725)	Exemestane (*n* = 310)	Letrozole (*n* = 1,202)
Gender, *n* (%)				
Female	2,164 (96.74)	700 (96.55)	299 (96.45)	1,165 (96.92)
Male	17 (0.76)	12 (1.66)	0 (0.00)	5 (0.42)
Unknown	56 (2.50)	13 (1.79)	11 (3.55)	32 (2.66)
Weight (kg), *n* (%)				
<80	1,120 (50.07)	362 (49.93)	157 (50.65)	601 (50.00)
80–100	354 (15.82)	100 (13.79)	39 (12.58)	215 (17.89)
>100	37 (1.65)	17 (2.34)	3 (0.97)	17 (1.41)
Unknown	726 (32.45)	246 (33.93)	111 (35.81)	369 (30.70)
Median (kg)	68 (23.04–176)	68 (23.04–176)	69 (25.10–150)	68 (27.78–132)
Age (years), *n* (%)				
<18	32 (1.43)	24 (3.31)	0 (0.00)	8 (0.67)
18–44	60 (2.68)	23 (3.17)	17 (5.48)	20 (1.66)
45–65	616 (27.54)	232 (32.00)	98 (31.61)	286 (23.79)
>65	759 (33.93)	286 (39.45)	119 (38.39)	354 (29.45)
Unknown	770 (34.42)	160 (22.07)	76 (24.52)	534 (44.43)
Median (years)	66 (1–93)	66 (3.33–89)	66 (25–93)	66 (1–93)
Occupation of reporters, *n* (%)				
Consumer (CN)	511 (22.84)	220 (30.34)	85 (27.42)	206 (17.14)
Physician (MD)	981 (43.85)	231 (31.86)	126 (40.65)	624 (51.91)
Pharmacist (PH)	125 (5.59)	41 (5.66)	24 (7.74)	60 (4.99)
Other health-professional (OT)	411 (18.37)	85 (11.72)	58 (18.71)	268 (22.30)
Unknown	209 (9.34)	148 (20.41)	17 (5.48)	44 (3.66)
Reported countries, *n* (%)				
US	585 (26.15)	348 (48.00)	100 (32.26)	137 (11.40)
Non-US	1,596 (71.35)	351 (48.41)	203 (65.48)	1,042 (86.69)
Unknown	56 (2.50)	26 (3.59)	7 (2.26)	23 (1.91)
Outcomes, *n* (%)				
Death (DE)	116 (5.19)	31 (4.28)	10 (3.23)	75 (6.24)
Disability (DS)	77 (3.44)	34 (4.69)	13 (4.19)	30 (2.50)
Hospitalization (HO)	504 (22.53)	115 (15.86)	66 (21.29)	323 (26.87)
Life-threatening (LT)	66 (2.95)	20 (2.76)	11 (3.55)	35 (2.91)
Other serious (Important medical event, OT)	1,102 (49.26)	308 (42.48)	153 (49.35)	641 (53.33)
Required intervention to prevent permanent impairment/damage (RI)	10 (0.45)	9 (1.24)	0 (0.00)	1 (0.08)
Unknown	362 (16.18)	208 (28.69)	57 (18.39)	97 (8.07)

Notes: Continuous numerical variables are expressed as mean ± standard deviation, and categorical variables are presented as n (%).

### 3.2 Different AI-related signals

Our analysis highlights the occurrence of dermatologic AEs across different AI treatment regimens. As shown in Table 2, both anastrozole (ROR, 1.07; 95% CI, 1.03–1.11) and exemestane (ROR, 1.10; 95% CI, 1.04–1.16) demonstrated significant signals associated with dermatologic AEs, suggesting a strong link with dermatologic toxicity. In contrast, letrozole (ROR, 0.94; 95% CI, 0.91–0.98) showed no positive association with dermatologic AEs.

**TABLE 2:** Signal strength of reports of dermatologic adverse events related to aromatase inhibitors in FAERS database.

Aromatase inhibitors	The report number	ROR (95%CI)	PRR (χ^2^)	EBGM (EBGM05)	IC (IC_025_)
Anastrozole	2,986	1.07 (1.03–1.11)	1.07 (12.71)	1.07 (1.03)	0.09 (−1.57)
Exemestane	1,331	1.1 (1.04–1.16)	1.1 (11.7)	1.1 (1.05)	0.13 (−1.53)
Letrozole	3,975	0.94 (0.91–0.98)	0.95 (12.29)	0.95 (0.92)	−0.08 (−1.74)

Note: ROR, reporting odds ratio; CI, confidence interval; PRR, proportional reporting ratio; χ2, chi-squared; IC, information component; IC_025_, the lower limit of 95%CI, of the IC; EBGM, empirical Bayesian geometric mean; EBGM05, the lower limit of 95% CI, of EBGM.

### 3.3 The spectrum of dermatologic adverse effects at the preferred term (PT) levels

**FIGURE 2:**
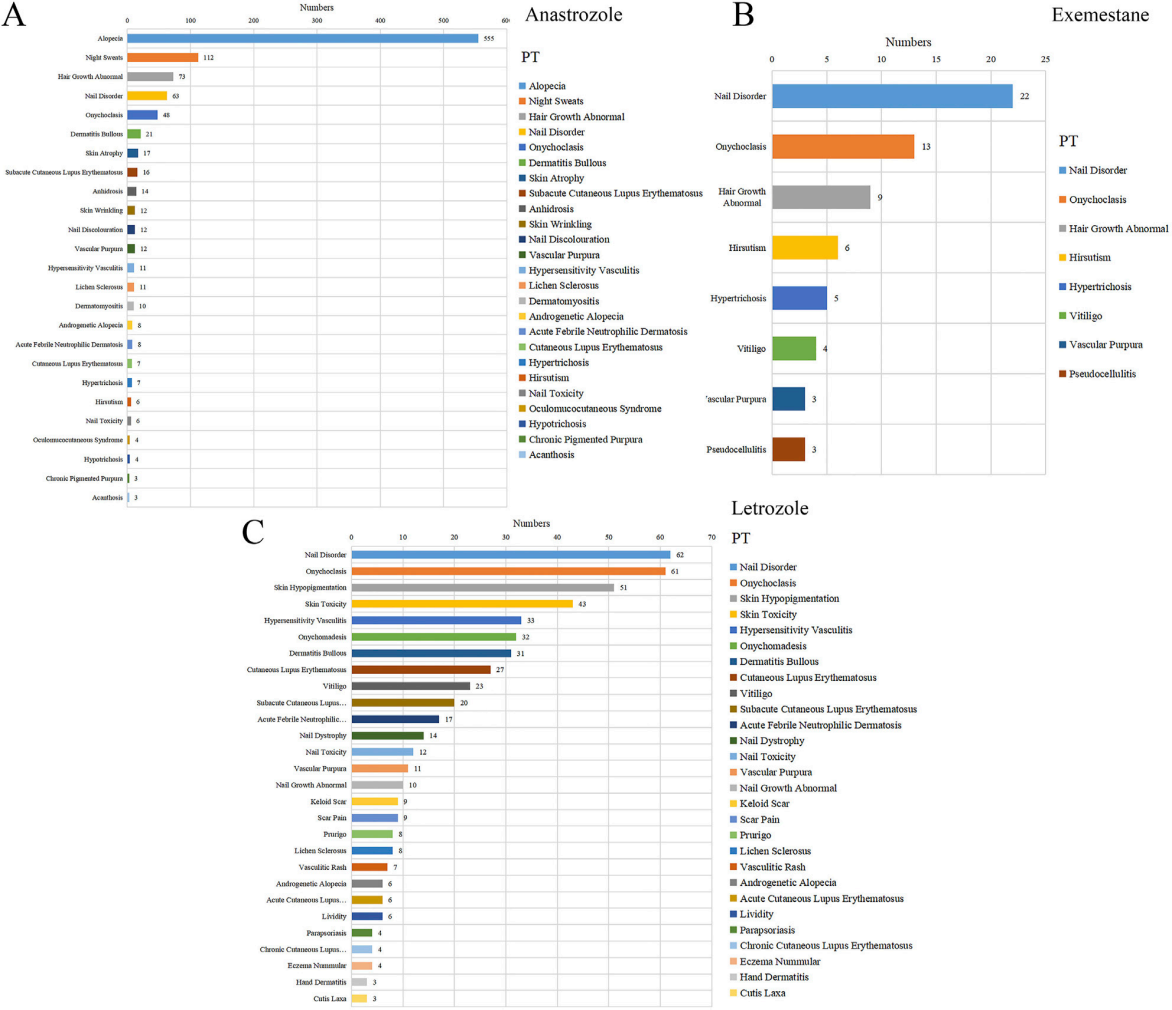
The statistics for different PTs in reports with dermatologic adverse events associated with several aromatase inhibitors. **(A)** Anastrozole, **(B)** Exemestane, **(C)** Letrozole.

A total of 61 positive PT-level signals were identified (Figure 2). Significant PT signals were observed for three selected AI agents: anastrozole (25 PTs), exemestane (8 PTs), and letrozole (28 PTs). Anastrozole was strongly associated with dermatologic alopecia (*n* = 555), night sweats (*n* = 112), and abnormal hair growth (*n* = 173). For exemestane, nail disorder (*n* = 22), onychoclasis (*n* = 13), and abnormal hair growth (*n* = 9) were prominent signals. Notably, nail disorder (*n* = 22) and onychoclasis (*n* = 13) were the top two signals for letrozole among its 28 positive PTs, showing a marked association across all three AIs.

**FIGURE 3:**
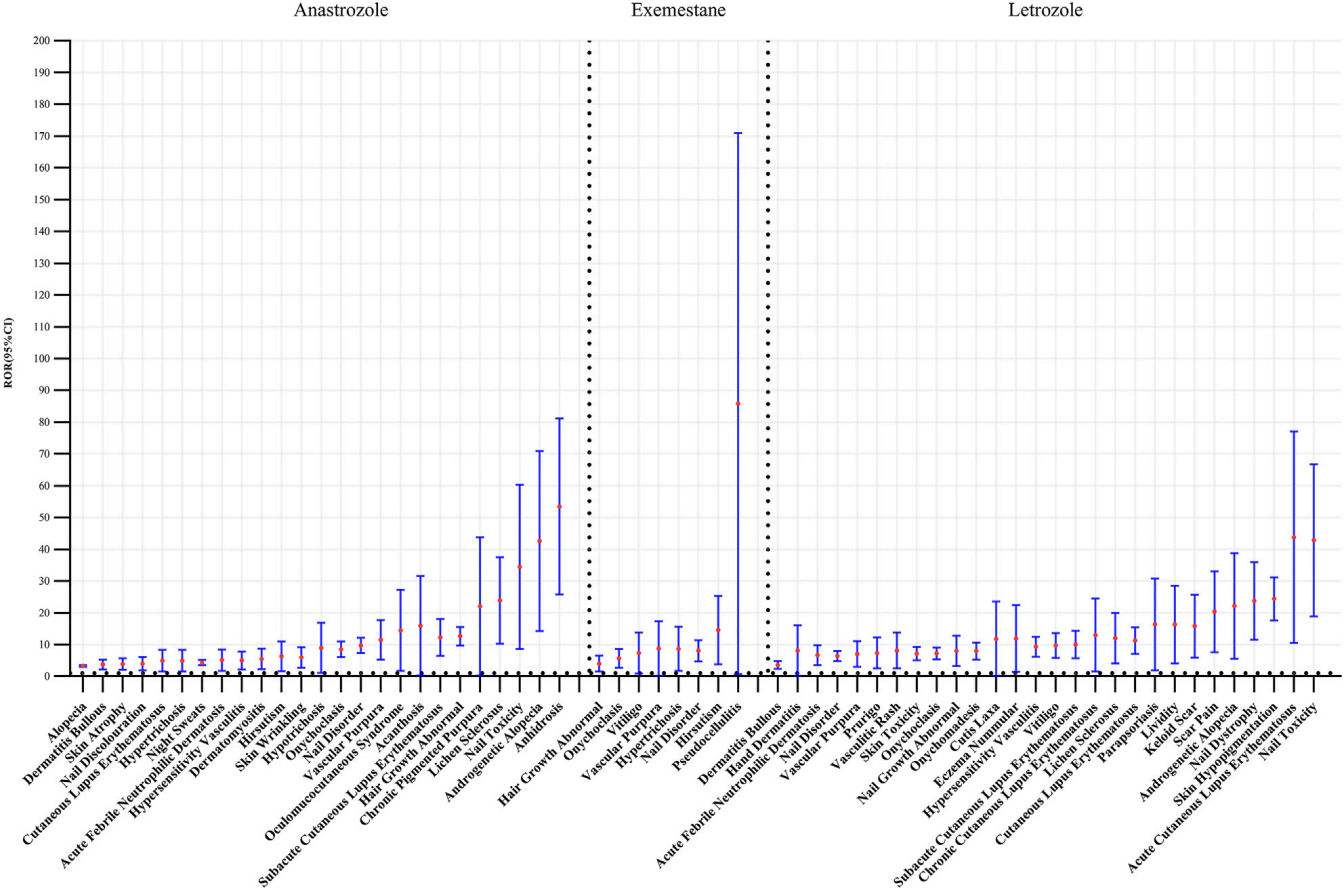
Forest plots of different aromatase inhibitors agents inducing dermatologic toxicity.

Disproportionality analysis on these PTs, indicated by ROR values > 1, confirmed a significant association between AIs and skin-related adverse reactions. Specifically, 25 positive signals (ROR = 1.07, 95% CI = 1.03–1.11) for anastrozole, 8 signals (ROR = 1.1, 95% CI = 1.04–1.16) for exemestane, and 28 signals (ROR = 0.94, 95% CI = 0.91–0.98) for letrozole were detected. As shown in Figure 3, the highest-ranked associations were anhidrosis for anastrozole (ROR = 48.68, CI = 28.48–83.22), pseudo cellulitis for exemestane (ROR = 57.73, CI = 18.36–181.48), and nail toxicity for letrozole (ROR = 38.40, CI = 21.47–68.68).

Logistic regression analysis (Table 3) indicated that hospitalization and age were significant protective factors for dermatologic AEs associated with anastrozole and exemestane (*P* < 0.05). However, body weight was not a significant factor for dermatologic AEs (*P* > 0.05).

**TABLE 3:** Multivariate logistic regression model of dermatologic adverse events.

Characteristic	Number	OR (95%CI)	*P*-value
Anastrozole			
Hospitalization			
No	3,478	Reference	
Yes	1,087	0.46 (0.32–0.65)	<0.001
Age			
<65	2,311	Reference	
≥65	2,254	1.51 (1.18–1.93)	0.001
Weight			
50–100 kg	4,137	Reference	
<50 or >100 kg	428	0.73 (0.44–1.15)	0.200
Exemestane			
Hospitalization			
No	1,399	Reference	
Yes	839	0.35 (0.21–0.55)	<0.001
Age			
<65	1,105		
≥65	1,133	1.56 (1.06–3.00)	0.024
Weight			
50–100 kg	1961	Reference	
<50 or >100 kg	277	0.50 (0.22–0.98)	0.062

Abbreviation: OR, odds ratio; CI, confidence interval; *P* < 0.05 were considered statistically significant.

### 3.4 TTO analysis of dermatologic AEs by AIs

**FIGURE 4:**
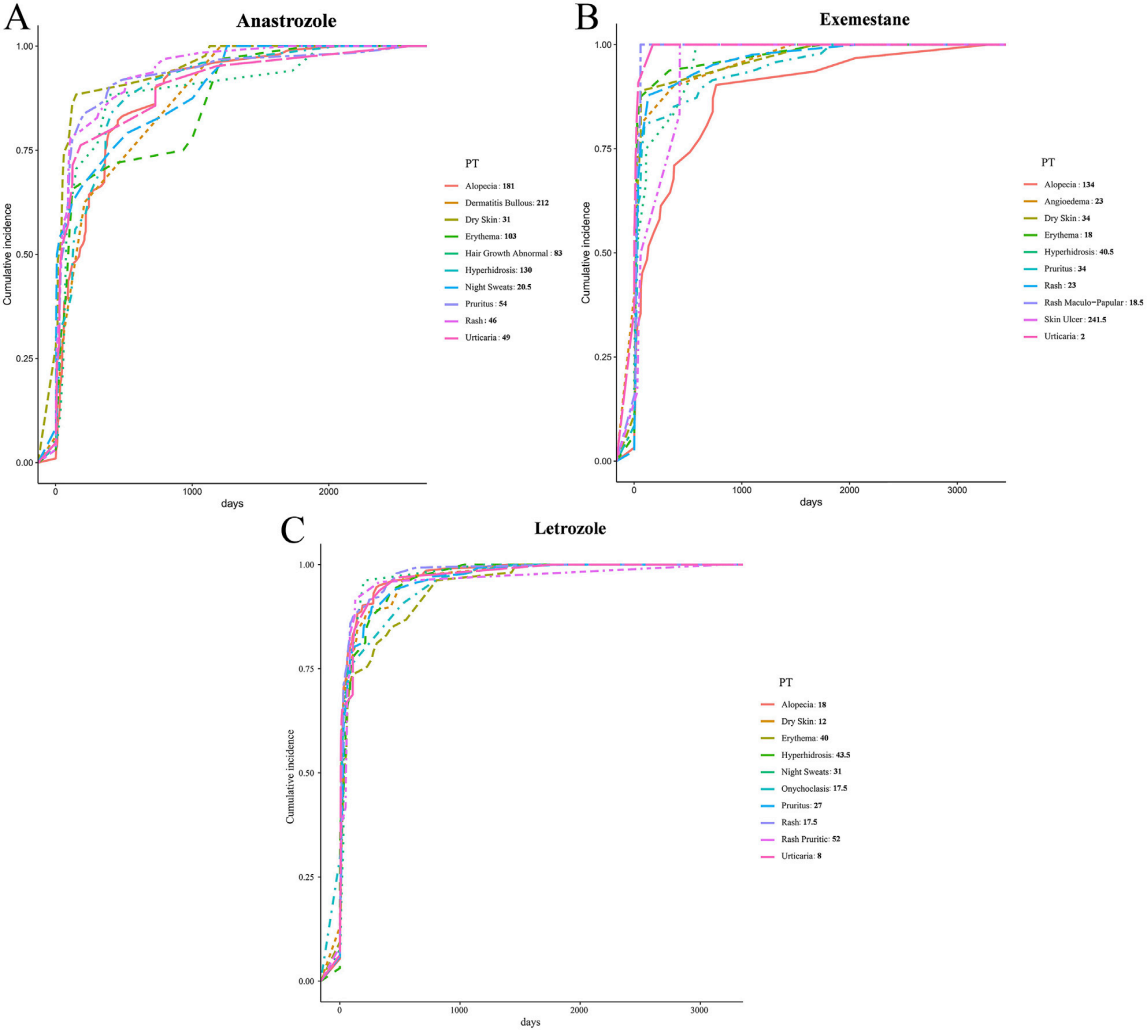
Time-to-onset on dermatologic AEs elicited by AIs. **(A)** Anastrozole, **(B)** Exemestane, **(C)** Letrozole.

The onset times for 10 PTs (alopecia, bullous dermatitis, dry skin, erythema, abnormal hair growth, hyperhidrosis, night sweats, pruritus, rash, and urticaria) were significantly associated with anastrozole. Among these, night sweats had the shortest median onset time at 20.5 days, while bullous dermatitis had the longest at 212 days. In the letrozole group, 10 PTs (alopecia, angioedema, dry skin, erythema, hyperhidrosis, pruritus, rash, maculopapular rash, skin ulcer, and urticaria) were observed. Urticaria showed the shortest onset time with a median of 2 days, and skin ulcer had the longest at 241.5 days. For exemestane, onset times were noted for alopecia, dry skin, erythema, hyperhidrosis, night sweats, onychoclasis, pruritus, rash, pruritic rash, and urticaria. Urticaria presented the shortest onset time at a median of 8 days, and pruritic rash showed the longest at 52 days. Onset times for all PTs are shown in Figure 4.

## 4 Discussion

AIs, categorized as nonsteroidal and steroidal, are widely used as adjuvant therapy and represent the gold standard for estrogen receptor (ER)+ BC management. Among the nonsteroidal AIs, anastrozole and letrozole significantly benefit ER + BC patients by inhibiting aromatase activity through multiple mechanisms (Fusi et al., 2014; Jameera Begam et al., 2017). For example, a clinical trial involving 3,864 postmenopausal women with BC demonstrated that anastrozole provided greater clinical benefits than placebo, underscoring its therapeutic value (Wollina et al., 2018). Additionally, the combination of ribociclib with letrozole has shown substantial efficacy, with a median PFS of 21.8 months (95% CI, 13.9–25.3) (Fasching et al., 2024). Exemestane, the only third-generation steroidal AI, is also clinically beneficial for postmenopausal BC patients (Jameera Begam et al., 2017). The frequent clinical use of anastrozole, exemestane, and letrozole warrants examination in this study. However, the dermatologic risks associated with AIs cannot be overlooked. For instance, a phase 3b study of ribociclib in BC reported a 35.1% incidence of alopecia with letrozole treatment. Additional dermatologic adverse effects, such as hot flashes, pruritus, and rash, have been noted in the literature (Fasching et al., 2024). These adverse dermatologic events not only impact patients’ quality of life but may also lead to interruptions or even discontinuation of cancer therapy (Sussman et al., 2019). While the benefits of AIs generally outweigh the risks, proactive prevention strategies are essential. This study conducts a comprehensive assessment of dermatologic risks associated with the commonly used AIs—exemestane, anastrozole, and letrozole—over the past 2 decades, utilizing real-world data from an AE reporting database.

In our study, the relatively low ROR for dermatologic AEs associated with letrozole (ROR = 0.94) compared to anastrozole and exemestane may be attributed to several pharmacokinetic and pharmacodynamic differences. Letrozole has a longer half-life (approximately 4 days) and reaches steady-state plasma concentrations over a longer period (60 days) (Buzdar et al., 2002) compared to anastrozole and exemestane, which have shorter half-lives (1–2 days) and quicker peak drug levels (Hamadeh et al., 2018). This steady accumulation may result in fewer fluctuations in drug levels, potentially reducing the incidence of acute skin reactions. Therefore, letrozole’s pharmacokinetic profile, with a longer half-life and stable plasma concentrations, might lead to a more gradual onset of adverse effects, including dermatologic reactions. Additionally, each AI is metabolized via different pathways, which can influence the formation of reactive metabolites or drug–drug interactions. Letrozole is primarily cleared by CYP2A6 (with minor involvement of CYP3A4), and it notably inhibits CYP2A6 itself, potentially leading to self-limited metabolism and more stable drug levels over time (De Placido et al., 2018). In contrast, anastrozole is mainly metabolized by CYP3A4 (with contributions from CYP3A5, CYP2C8, and glucuronidation), and it can weakly inhibit several CYP enzymes (e.g., 1A2, 2C8/9, 3A4) (De Placido et al., 2018). Exemestane undergoes oxidation by CYP3A4 and reduction by CYP450 enzymes, including 11β-hydroxylation via CYP4A11 (De Placido et al., 2018). Given this metabolic profile, letrozole’s unique CYP2A6 pathway, combined with its steady accumulation and lack of active metabolites, may make it less prone to certain immune-mediated skin reactions or erratic plasma fluctuations that could precipitate rashes. This could partly explain why letrozole has a lower ROR for dermatologic issues relative to anastrozole and exemestane.

Recent clinical publications have highlighted the dermatologic toxicities induced by aromatase inhibitors (AIs) (Wilkinson and Hardman, 2017), Moreover, AIs have been shown to influence humoral immunity and immunoglobulin levels (Cutolo et al., 2012; Aguilar-Pimentel et al., 2020). Increasing evidence suggests that reduced estrogen levels can provoke hyperactive neutrophils, which adhere to vascular endothelium, potentially triggering autoimmune vasculitis and adverse dermatologic responses (Zarkavelis et al., 2016; Woodford et al., 2019). Estrogen thus plays a critical role in preserving skin vasculature and function. Although AIs benefit postmenopausal women with BC by reducing estrogen, excessive estrogen deficiency adversely impacts skin maintenance. Consequently, AIs in clinical use often leads to dermatologic side effects such as hot flushes, sweating, vaginal dryness, nail disorders, onychoclasis, hypopigmentation, and vascular purpura (Pagani et al., 2014; Fasching et al., 2024; Zhang et al., 2024). The prevalence of these adverse effects calls for rigorous dermatologic monitoring to ensure safe AI application.

Alopecia is particularly common in BC patients treated with AIs, with a notable incidence among AEs (Fasching et al., 2024). Alopecia affects approximately 2.5% of BC patients, and about 8% discontinue AI therapy due to this adverse effect (Freites-Martinez et al., 2018; Ferreira et al., 2019; Behbahani et al., 2021). In our retrospective pharmacovigilance analysis, 76.55% of alopecia cases were associated with anastrozole (555 out of 725 reports). All three AIs were linked to hair abnormalities, with alopecia and abnormal hair growth linked to anastrozole, androgenetic alopecia linked to letrozole, and abnormal hair growth linked to exemestane. These findings predict a high risk of alopecia with AIs in BC therapy, suggesting that concurrent use of AIs with minoxidil may help sustain hair follicle health (Freites-Martinez et al., 2018; Ferreira et al., 2019).

Our analysis found positive signals for onychoclasis, vascular responses (e.g., hypersensitivity vasculitis, vascular purpura), hair abnormalities (e.g., alopecia, androgenetic alopecia, hair growth abnormality), and nail toxicity (e.g., nail disorder, discoloration, nail dystrophy) across all three AIs, indicating a need for appropriate patient care. Additionally, dry skin, erythema, hyperhidrosis, pruritus, and rash were frequently associated with AI-related skin impairments, emphasizing the necessity for concurrent protective strategies during AI treatment in BC patients. While letrozole showed a negative association with skin-related AEs (ROR 0.94; 95% CI 0.91–0.98), anastrozole (ROR 1.07; 95% CI 1.03–1.11) and exemestane (ROR 1.1; 95% CI 1.04–1.16) had significant positive associations, underscoring the need for close monitoring of dermatologic adverse effects with these drugs.

Regarding onset time, all three AIs were associated with urticaria, with a median onset of just 2 days for exemestane, indicating a rapid adverse response. Most positive PTs had onset times of less than 50 days (dry skin, alopecia, erythema, night sweats, onychoclasis, pruritus, rash, urticaria, angioedema, and maculopapular rash), suggesting acute impacts on patient quality of life. These findings highlight the need for proactive intervention, enabling targeted measures based on onset timing.

This pharmacovigilance study has limitations. First, duplicate reports, false information, and missing data in the FAERS database reduce data accuracy. For example, reports with inaccurate dates were excluded, though they may have contained relevant information on AI-related dermatologic AEs. Second, FAERS data are based on voluntary submissions, primarily from healthcare providers, which lack direct causality. This limitation is particularly relevant when interpreting discrepancies between our findings (e.g., the relatively low ROR for letrozole) and prior literature, as FAERS cannot establish a direct causal relationship between AIs and dermatologic AEs. Third, this study reflects only FAERS reports, suggesting an elevated risk of AEs with AIs but not a causal relationship with dermatologic effects. Finally, FAERS is a spontaneous global reporting system that introduces selection bias. Most reports in this study originated from the United States, limiting geographical diversity in the data.

## 5 Conclusion

Given the high incidence of AI-induced dermatologic toxicity in postmenopausal women with BC treatment, tailored management strategies should be prioritized the specific dermatologic impact of each AI. Our analysis is the first to highlight the global prevalence of dermatologic adverse reactions associated with AIs, emphasizing that these AEs are a critical factor in AI utilization. Addressing drug-induced skin disorders is essential to ensure the broader application of AIs. This chapter calls for further research into AI-related dermatologic damage in BC management to optimize patient care comprehensively. For high-risk patients, especially those with pre-existing dermatologic conditions, close monitoring is recommended to detect skin-related AEs early and allow for timely intervention. Regular assessments for dryness, alopecia, and rashes, combined with patient education on managing these issues, will help maintain quality of life during treatment.

## Data Availability

Publicly available datasets were analyzed in this study. This data can be found here: https://www.fda.gov/drugs/questions-and-answers-fdas-adverse-event-reporting-system-faers/fda-adverse-event-reporting-system-faers-public-dashboard.

## Abbreviations

BC, breast cancer; AIs, aromatase inhibitors; PFS, progression-free survival; AE, adverse event; FAERS, FDA Adverse Event Reporting System; SOC, system organ class; ROR, reporting odds ratio; PRR, proportional reporting ratio; EBGM, empirical Bayes geometric mean; BCPNN, Bayesian confidence propagation neural network; TTO, time to onset; CI, confidence interval; PT, preferred term; ER, estrogen receptor; EBGM05, the lower limit of 95% CI of EBGM. IC, information component; IC025, the lower limit of 95%CI of the IC; χ2, chi-squared.
