# Supplementary material for: Evaluation of dermatologic adverse events associated with aromatase inhibitors: insights from the FAERS database
Source: Front Pharmacol. 2025 May 14;16:1529342. doi: 10.3389/fphar.2025.1529342 (PMC12116566; doi:10.3389/fphar.2025.1529342)
Supplement: Supplementary file 1 [file Table1.docx]

**Table S1. 2×2 table for signal detection.**

| **Type of drug** | **Target adverse** **reaction reports** | **Other adverse reaction reports** | **Sum** |
| --- | --- | --- | --- |
| **Target drug** | a | b | a+b |
| **Other drugs** | c | d | c+d |
| **Sum** | a+c | b+d | N=a+b+c+d |

**a**, Number of reports containing both the target drug and target adverse reaction reports; **b**, Number of reports containing other adverse reaction reports of the target drug; **c**, Number of reports containing the target adverse reaction reports of other drugs; **d**, Number of reports containing other drugs and other adverse reaction reports; **N**, The number of reports.
