# Supplementary material for: Evaluation of dermatologic adverse events associated with aromatase inhibitors: insights from the FAERS database
Source: Front Pharmacol. 2025 May 14;16:1529342. doi: 10.3389/fphar.2025.1529342 (PMC12116566; doi:10.3389/fphar.2025.1529342)
Supplement: Supplementary file 2 [file Table2.docx]

**Table S2. Four major algorithms applied for signal detection.**

| **Algorithms** | **Equation** | **Criteria** |
| --- | --- | --- |
| **ROR** | ROR=(a/c)/(b/d) | 95% CI>1, N≥3 |
|  | 95%CI=e^ln(ROR)±1.96(1/a+1/b+1/c+1/d)^0.5^ |  |
| **PRR** | PRR=[a/(a+b)]/[c/(c+d)] | PRR≥2, χ^2^≥4, N≥3 |
|  | χ^2^=[(ad-bc)^2](a+b+c+d)/[(a+b)(c+d)(a+c)(b+d)] |  |
| **MGPS** | EBGM=a(a+b+c+d)/[(a+c)(a+b)] | EBGM05>2 |
|  | EBGM05=e^ln(EBGM)-1.64(1/a+1/b+1/c+1/d)^0.5^ |  |
| **BCPNN** | IC=log_2_a(a+b+c+d)/[(a+c)(a+b)] | IC025>0 |
|  | IC025=e^ln(IC)−1.96(1/a+1/b+1/c+1/d)^0.5^ |  |

**a**, Number of reports containing both the target drug and target adverse reaction reports; **b**, Number of reports containing other adverse reaction reports of the target drug; **c**, Number of reports containing the target adverse reaction reports of other drugs; **d**, Number of reports containing other drugs and other adverse reaction reports. **95%CI**, 95% confidence interval; **N**, the number of reports; **χ^2^**, chi-squared; **EBGM**, empirical Bayesian geometric mean; **EBGM05**, the lower limit of 95% CI of EBGM; **IC**, information component; **IC025**, the lower limit of 95% CI of the IC. **ROR**, reporting odds ratio; **PRR**, proportional reporting ratio; **MGPS**, multi-item gamma poisson shrinker; **BCPNN**, bayesian confidence propagation neural network.
